# Forme précoce du syndrome de Wilkie: complication rare de la chirurgie pour scoliose à propos d’un cas et revue de la littérature

**DOI:** 10.11604/pamj.2016.25.90.8773

**Published:** 2016-10-17

**Authors:** Mamadou Mour Traore, Pape Alassane Leye, Mamadou Diawo Bah, Charles Valérie Alain Kinkpe, Pape Ibrahima Ndiaye, Mohamed Daffe, Alpha Omar Toure, Oumar Kane

**Affiliations:** 1 Centre Hospitalier de l’Ordre de Malte à Dakar, Sénégal; 2 Service d’Anesthésie - Réanimation CHU Aristide Le Dantec à Dakar, Sénégal; 3 Département d’Anesthésie - Réanimation CHU Fann à Dakar, Sénégal; 4 Service de Chirurgie Générale CHU Aristide Le Dantec à Dakar, Sénégal; 5 Département d’Anesthésie-Réanimation CHU Fann à Dakar, Sénégal

**Keywords:** Artère mésentérique supérieure, occlusion duodénale, scoliose idiopathique, Superior mesenteric artery, duodenal obstruction, idiopathic scoliosis

## Abstract

Le syndrome de la pince aorto-mésentérique est une complication rare pouvant survenir après une correction chirurgicale de scoliose. Les phénomènes de traction verticale de l’artère mésentérique et le rétrécissement de l’angle aorto-mésentérique lors de la correction sont à l’origine de ce syndrome par compression du troisième duodénum. Nous rapportons ici l’observation d’un cas précoce de syndrome de la pince aorto-mésentérique secondaire à une correction chirurgicale d’une scoliose idiopathique chez une fille. Une jeune fille opérée d’une arthrodèse vertébrale postérieure pour une scoliose idiopathique avait présenté au troisième jour postopératoire des vomissements incoercibles avec arrêt des matières et des gaz. Le scanner abdominal réalisé en urgence avait permis le diagnostic d’un syndrome de l’artère mésentérique supérieure. La prise en charge consistait à une mise au repos du tube digestif associé à une nutrition parentérale précoce et une correction des déséquilibres hydroélectrolytiques. Devant l’absence d’amélioration clinique, l’indication opératoire fut posée. L’évolution a été favorable avec des suites opératoires simples et une reprise alimentaire orale à quatrième jour, une sortie à domicile au septième jour postopératoire.Les facteurs favorisants sont les sujets jeunes, de morphotype longiligne avec un faible IMC inférieur à 18.Les signes scannographiques sont une dilatation gastrique importante avec un arrêt net au niveau de 3ème duodénum. La prise en charge est multidisciplinaire étant médicale d’abord et chirurgicale en cas d’échec. Une meilleure connaissance des facteurs prédictifs d’échec du traitement médical permettrait de réduire la durée de séjour hospitalier.

## Abstract (en)

Superior mesenteric artery syndrome is a rare complication occurring after surgical treatment for scoliosis. Surgical correction of the scoliotic spine produces vertical traction on the mesenteric artery and the narrowing of the aortomesenteric angle, hence the compression of the third part of the duodenum causing the syndrome. We here report the case of a young girl with an early form of superior mesenteric artery syndrome secondary to surgical correction of idiopathic scoliosis. The patient underwent posterior spinal fusion for idiopathic scoliosis. On the third postoperative day she experienced uncontrollable vomiting associated with no evacuation of faeces and no passing of flatus. Urgent abdominal CT scan was performed which allowed the diagnosis of superior mesenteric artery syndrome. Treatment consisted in the rest of the digestive tract associated with early parenteral nutrition and correction of fluid and electrolyte imbalances. Lacking evidence of clinical improvement, surgical indication was posed. The evolution was favourable with an uneventful postoperative recovery and the resumption of adequate food intake on the fourth postoperative day. The patient was discharged on the seventh postoperative day. Contributing factors are young age, long-limbed morphotype with BMI below 18. CT scan shows an important gastric dilatation associated with complete halt of the third part of the duodenum. The treatment is multidisciplinary, medical (first-line treatment) and surgical (if medical treatment do not help). A better knowledge of the predictors of medical treatment failure would reduce the length of hospital stay.

## Introduction

Le syndrome de la pince aorto-mésentérique ou syndrome de l’artère mésentérique est une occlusion duodénale secondaire à une compression extrinsèque du troisième duodénum par l’artère mésentérique supérieure en avant et l’aorte en arrière. Il résulte d’une véritable compression d’origine vasculaire du duodénum par fermeture de l’angle formé par l’aorte et l’artère mésentérique supérieure [1]. Plusieurs circonstances sont à l’origine de ce syndrome: perte de poids rapide (anorexie mentale, brulure étendue, syndrome de malabsorption) diminuant le tissu graisseux protégeant le duodénum, hématome rétropéritonéal, utérus gravide ou correction d’une déformation du rachis par contention plâtrée ou par la chirurgie. La correction de la cyphose est le facteur déterminant en entrainant la fermeture de l’angle aorto-mésentérique [2]. Nous rapportons un cas précoce de syndrome de l’artère mésentérique suite à la correction d’une scoliose idiopathique au Sénégal. Une revue de la littérature a permis d’optimiser la prise en charge.

## Patient et observation

**Figure 1:**
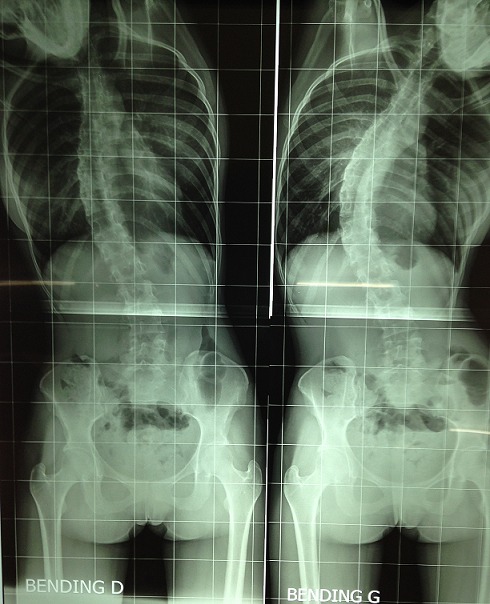
Clichés radiographiques préopératoires montrant la scoliose et le morphotype

**Figure 2:**
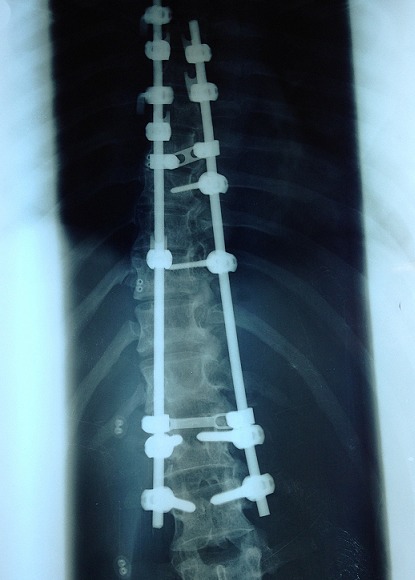
Images d’arthrodèse postérieure T4-L2 avec l’instrumentation utilisée

**Figure 3:**
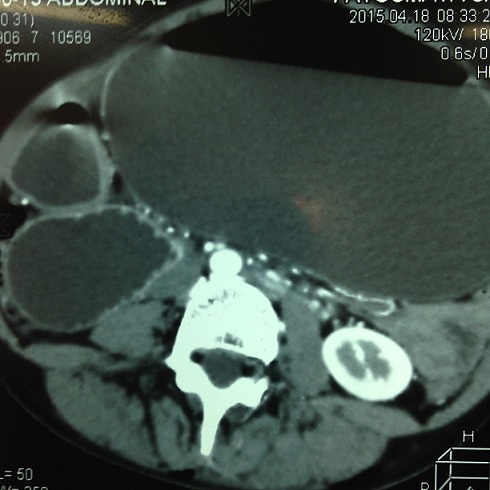
Dilatation gastrique majeure secondaire à l’occlusion du 3^ème^ème duodénum

Après l’obtention du consentement de la patiente et l’accord du comité d’éthique national. Une jeune femme âgée de 25ans, suivie dans notre service pour une scoliose dorsale idiopathique était reçue en consultation d’anesthésie à deux semaines d’une arthrodèse par voie postérieure. L’interrogatoire n’avait décelé aucun antécédent pathologique particulier personnel et familial. L’examen notait un poids à 40kg, une taille de 170cm (IMC= 13,84) avec un morphotype longiligne. Le bilan infectieux préopératoire ne révélait aucune anomalie (GB=6700, CRP <6mg/l, ECBU normal) et les clichés radiographiques comprenaient un full spine face/profil et des clichés de bending D/G (Figure 1). L’angle de Cobb mesurée était de 56°. Le traitement chirurgical avait consisté à une arthrodèse vertébrale par voie postérieure sur plusieurs étages vertébraux allant de T4 - L2 (Figure 2). L’intervention était réalisée sous anesthésie générale avec intubation orotrachéale par une sonde armée associée à une rachianalgésie à la morphine en raison de 10ug/kg soit 400ug au total. L’induction après administration de 1g de cefuroxime, était faite au propofol, fentanyl et vecuronium et l’entretien par de l’isoflurane et des réinjections de fentanyl et de vecuronium. La technique de l’hypotension artérielle contrôlée (cathéter artériel) avec de l’isoflurane associée à du propofol en administration continue était réalisée jusqu’à la mise en place du matériel. L’arthrodèse était effectuée avec du matériel type SPINEWAY^®^ et mise en place des greffons vertébraux. L’intervention a duré 2h15min et le saignement peropératoire estimé à 500ml. La patiente était restée stable durant l’intervention sans aucun incident ni accident peropératoire. L’analgésie postopératoire était multimodale associant de la kétamine en bolus à l’induction puis en continu jusqu’à j2, du paracétamol 3g/24h, du kétoproféne 100mg/24h. La prévention des nausées vomissement postopératoires et des troubles du transit comprenait du metopimazine, de ladexamethasone et de la trimébutine. La patiente après un séjour en SSPI était transférée en secteur d’hospitalisation le même jour. L’ablation de la sonde naso-gastrique (SNG) était réalisée au réveil en SSPI.L’alimentation liquide et chaude était débutée dès la huitième heure postopératoire. Du premier jour jusqu’au deuxième jour elle présentait plusieurs épisodes de vomissement et un léger ballonnement abdominal. Cette symptomatologie a été mise sous le compte des effets adverses de la morphine dans un premier temps. A j4 les vomissements étaient devenus persistants associés à un abdomen très distendu et douloureux spontanément et à la palpation. Devant la persistance de ces symptômes l’avis des chirurgiens viscéraux a été recueilli permettant la réalisation d’un scanner à J5 et de poser le diagnostic de syndrome de la pince aorto-mésentérique devant des signes d’occlusion haute à type de dilatation majeure de l’estomac associée à une importante stase liquidienne (Figure 3) et une réduction de l’angle aorto-mésentérique à 7°.Devant cette dilatation majeure la SNG a été remise en place ramenant ainsi 3000ml de liquide biliaire d’emblée et atténuant considérablement la douleur et la distension abdominale. L’ionogramme sanguin montrait une hyponatrémie à 129 mmol/L et une hypokaliémie à 2,7mmol/L.

**Figure 4:**
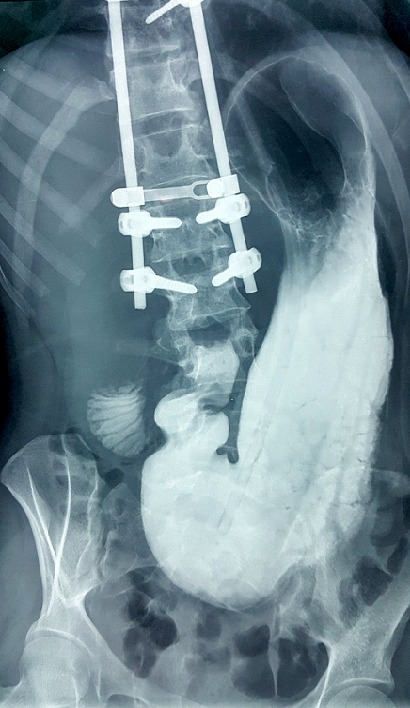
Occlusion nette au 3^ème^ duodénum au transit aux hydrosolubles

**Figure 5:**
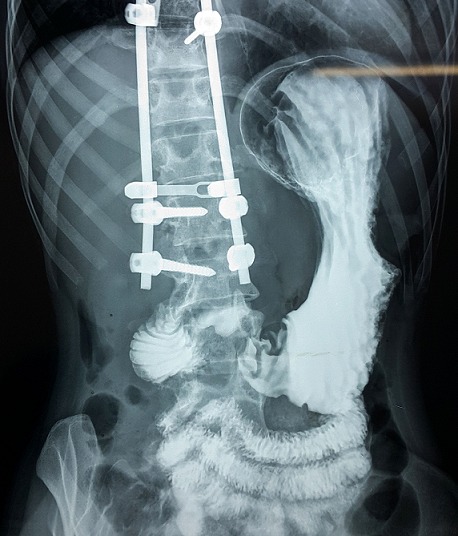
Contrôle satisfaisant de la continuité digestive après intervention (transit aux hydrosolubles)

La prise en charge consistait à une mise au repos du tube digestif par diète alimentaire avec mise en place de la SNG en siphonage et l’introduction d’une nutrition parentérale par voie veineuse centrale jugulaire interne. Ce traitement était associé à la correction des troubles ioniques et une analgésie à base d’antispasmodique et de néfopam. Les éléments de surveillance étaient la production liquidienne au niveau de la SNG, la palpation abdominale, le poids et la température corporels, une gazométrie artérielle quotidienne couplée à un ionogramme sanguin et un ASP. L’évolution a été marquée par un échec de la tentative de ré-nutrition orale après ablationde la SNG à J11 avec réapparition des vomissements, de la douleur épigastrique et de la distension abdominale. Ce constat avait motivé un transit aux hydrosolubles réalisé à J13 concluant à un petit passage en aval de l’obstacle au niveau de D3 (Figure 4). L’intervention fut effectuée à J20 après stabilisation des équilibres hydro-électrolytiques et nutritionnels (natrémie à 140mmol/L, potassium à 3,7mmol/L, gazométrie normale, protidémie à 75g/l). L’acte chirurgical consistait à une dérivation par duodéno-jéjunostomie transmésocolique par une laparotomie médiane sus-ombilicale sous anesthésie générale et transfert en réanimation pour surveillance sur 48h. Après une semaine d’observation en milieu chirurgical d’ou un transit de contrôle avait montré un passage franc du produit de contraste dans le grêle sans fistule digestive (Figure 5), l’alimentation orale fut autorisée sans problème et la sortie décidée à J26 soit trois semaines de séjour hospitalier.

## Discussion

Le syndrome de la pince aorto-mésentérique a été publié par wilkie en 1927 sur une série de 75 malades d’ou le nom de syndrome de Wilkie [1]. Dans plusieurs travaux de la littérature ce syndrome de la pince aorto-mésentérique est décrit comme une complication rare pouvant survenir après une correction de scoliose [2–5].Cette affection est relatée dans la littérature avec des incidences variables allant de 0,5 à 4,7% [6]. Plusieurs études retrouvent une fréquence de 1% après une chirurgie du rachis [2]. Guigui et al dans une étude multicentrique prospective datant de 2005 et incluant 3311 patients estiment sa fréquence à 1/1000 [7]. En 1995, une revue de la littérature depuis 1970 a recensé 152 cas de syndrome de la pince aorto-mésentérique, parmi lesquelles la chirurgie pour scoliose était la deuxième cause avec 13,8%. L’importance du morphotype est identifiée comme un facteur de risque de survenu de ce syndrome. En effet selon Shah et al plus les sujets sont grands et minces plus ils risquent de fermer leur pince aorto-mésentérique après une correction chirurgicale postérieure ou par plâtre [8]. Un seul cas de syndrome de l’artère mésentérique supérieure après correction chirurgicale de scoliose chez une jeune fille obèse est rapporté par Walker et al [9]. Selon Altiok et al, l’indice de masse corporelle semble être un bon paramètre permettant d’identifier les patients à risque et au besoin de prévenir cette complication par un régime péri-opératoire adapté [10]. Certains auteurs préconisent une correction de cet indice en période préopératoire pour des patients ayant une déformation inférieure à 100°.

Dans la littérature, les symptômes de cette affection surviennent de façon tardive suivant un délai d’une semaine après l’intervention chirurgicale. Chez notre patient la survenue des signes d’occlusion haute était particulièrement précoce dès le troisième jour. Durant cette phase postopératoire précoce le diagnostic peut être rendu difficile par la survenue de nausées et vomissements postopératoires secondaires à l’utilisation de la morphine à forte dose pour l’analgésie postopératoire (titration morphine, rachianalgésie morphine). L’aggravation des épisodes de vomissement et l’apparition d’une distension abdominale au delà de 72h doivent faire rechercher ce syndrome particulièrement chez des sujets à risque. Il ressort dans la littérature que l’apparition des symptômes est toujours retardée d’au moins une semaine par rapport à la chirurgie [2]. Contrairement à cet argument, notre observation objective un syndrome de l’artère mésentérique particulièrement précoce à J4 de l’intervention. Dans la plupart des séries publiées le diagnostic est confirmé par un transit après une suspicion au scanner. Dans notre cas, les images scannographiques ont permis de poser le diagnostic devant le contexte périopératoire et la présence de facteur de risque (IMC < 18, morphotype longiligne). Cette présentation clinique du syndrome de la pince mésentérique démontre l’apport du scanner dans le diagnostic par rapport au transit aux hydrosolubles souvent mal toléré par le patient. Par ailleurs une dilatation majeure de l’estomac associée à une importante stase gastrique a une valeur de transit. Le diagnostic est également conforté par la mesure de l’angle de la pince qui était de 7° sur les clichés scannographiques avec injection de produit de contraste. D’après les données de la littérature, normalement cet angle est compris entre 45° et 60° alors qu’en cas de syndrome de la pince aorto-mésentérique, l’angle se ferme et ne mesure plus qu’entre 6° à 15° [1, 11, 12]. Il nous paraît important dès lors, que l’anesthésiste en charge de ces patients durant cette période péri-opératoire ne devrait pas ignorer cette complication chirurgicale. L’identification et la connaissance des facteurs de risque sont d’un grand intérêt surtout devant les nausées et vomissements postopératoires persistants en postopératoire au delà de 72h ou à distance de la chirurgie. Une attention particulière devrait être attaché durant cette phase aux analgésiques morphiniques très émétisants qui peuvent rendre le diagnostic déroutant. Les répercussions cliniques et biologiques de cette affection de par les vomissements incoercibles peuvent être graves et menacer le pronostic vital. La létalité de ce syndrome varie dans la littérature: en 1971, Evarts et al constataient 4 décès sur une série de 16cas ; en 1974, Hughes et al rapportaient 7 décès sur 37cas [13, 14]. Les causes principales étaient la déshydratation progressive et ses perturbations électrolytiques dues à un retard diagnostic. Les vomissements incoercibles sont à l’origine de troubles hydro-électrolytiques tels qu’une déshydratation, une hyponatrémie et une hypokaliémie. Ces anomalies biologiques ont été retrouvées chez notre patiente au moment du diagnostic précoce nécessitant une correction rapide.

La plupart des travaux de la littérature privilégient un traitement médical conservateur, la chirurgie n’est envisagée qu’en cas d’échec de celui-ci. Le traitement médical consiste à une mise au repos du tube digestif associée à une alimentation par voie parentérale et une correction des troubles hydro-électrolytiques durant plusieurs semaines [2, 11]. Cette option thérapeutique vise un gain pondéral pour le patient, favorable à un engraissement autour de l’artère mésentérique afin d’ouvrir l’angle de la pince [15, 16].Ce choix thérapeutique nécessite une observation du patient en milieu de surveillance continue en rapport aux nombreuses complications inhérentes à la nutrition artificielle au long court (thrombotiques, infectieuses, hydro-électrolytiques etc). Actuellement, le traitement médical est de plus en plus préconisé [9, 17, 18]. Son efficacité a été relatée par Altiok et al dans une série de 17 cas, un seul a nécessité un traitement chirurgical [9]. Cependant dans notre cas, le recours à la chirurgie (duodéno-jéjunostomie) était jugé nécessaire devant la persistance de la symptomatologie à l’ablation de la sonde gastrique après 20 jours de nutrition parentérale et de mise au repos du tube digestif. Selon Kadji et al le traitement chirurgical s’avère nécessaire dans 75% des cas [16]. Dans la littérature les facteurs prédictifs d’échec du traitement médical restent non élucidés. Il nous semble important d’identifier ces facteurs d’échec du traitement médical afin de proposer précocement la chirurgie dans le but de réduire le cout du traitement médical et la durée de séjour hospitalier. Chez notre patiente la chirurgie a permis de raccourcir la durée de séjour à une semaine après l’intervention contre 6 semaines pour le traitement conservateur [2]. Cependant le traitement médical trouve tout son intérêt dans l’optimisation de la prise en charge chirurgicale par une bonne préparation en corrigeant les troubles hydro électrolytiques et nutritionnels.

## Conclusion

Le syndrome de la pince aorto-mésentérique est une complication rare de la chirurgie de scoliose. Le diagnostic est suspecté devant des vomissements postopératoires persistants tardifs généralement au delà d’une semaine cependant la survenue peut être précoce. Les facteurs favorisants sont les sujets jeunes, de morphotype longiligne avec un faible IMC inférieur à 18.Les signes scannographiques sont une dilatation gastrique importante avec un arrêt net au niveau de 3^ème^ duodénum. La prise en charge est pluridisciplinaire (anesthésiste-réanimateur, chirurgien digestif, orthopédiste, radiologue). Le pronostic est généralement favorable sous réserve d’une prise en charge médico-chirurgicale rapide. Une meilleure connaissance des facteurs prédictifs d’échec du traitement conservateur permettrait de réduire le cout et la durée de séjour hospitalier.
